# The predictive value of mental health for long-term sickness absence: the Major Depression Inventory (MDI) and the Mental Health Inventory (MHI-5) compared

**DOI:** 10.1186/1471-2288-13-115

**Published:** 2013-09-17

**Authors:** Sannie Vester Thorsen, Reiner Rugulies, Pernille U Hjarsbech, Jakob Bue Bjorner

**Affiliations:** 1 The Danish National Research Centre for the Working Environment, Lersø Parkallé 105, DK-2100, Copenhagen, Denmark; 2 QualityMetric, 24Albion Road, Lincoln, Rl 02865, USA

**Keywords:** Head-to-head comparison, Survey instrument, Questionnaire

## Abstract

**Background:**

Questionnaires are valuable for population surveys of mental health. Different survey instruments may however give different results. The present study compares two mental health instruments, the Major Depression Inventory (MDI) and the Mental Health Inventory (MHI-5), in regard to their prediction of long-term sickness absence.

**Method:**

Questionnaire data was collected from N = 4153 Danish employees. The questionnaire included the MDI and the MHI-5. The information of long-term sickness absence was obtained from a register. We used Cox regression to calculate covariance adjusted hazard ratios for long-term sickness absence for both measures.

**Results:**

Both the MDI and the MHI-5 had a highly significant prediction of long-term sickness absence. A one standard deviation change in score was associated with an increased risk of long-term sickness absence of 27% for the MDI and 37% for the MHI-5. When both measures were included in the same analysis, the MHI-5 performed best.

**Conclusion:**

In general population surveys, the MHI-5 is a better predictor of long-term sickness absence than the MDI.

## Background

Mental disorder is a main cause of the disease burden in developed countries [1]. Thus, policy makers have an interest in the level of mental health and prevalence of mental health problems in the population. Clinical interviews are the gold standard for measuring psychiatric morbidity, however such interviews are costly. An alternative is self-report questionnaires of mental health. These questionnaires are cheaper and practical for population surveys and they can provide an assessment of all respondents. Questionnaire data on mental health has been shown to be associated with reduced workability [2,3], lower work performance, and sickness absence [4-7]. Questionnaires can, however, not find true prevalence but only estimate risk proportions [8].

Several mental health instruments exist and depending on the chosen questionnaire the estimate of the risk proportion of the population may be very different: For example, in Croatia, Rukavinia et al. used the Mental Health Inventory (MHI-5) and estimated that the proportion with ‘psychological distress’ (scored from 0 to 100, cut-off at 52 point) was 34% in the adult population [9]. In Canada the proportion with ‘high psychological distress’ was 38% using the K-10 scale (scored from 0 to 40, cut-off at 9 point) [10]. In Denmark, the risk proportion of ‘all kinds of depression’ was 7.1% using the Major Depression Inventory (MDI) (scored from 0 to 50, cut-off at 20 points) [11].

In the present study, we evaluate the two mental health instruments the Major Depression Inventory (MDI) and the Mental Health Inventory (MHI-5) in regard to the prediction of long-term sickness absence. If the mental health of a person causes him or her to have a long-term sickness absence period from work, it is a severe health problem. Furthermore, the cumulative cost of sickness absence due to mental health problems is an economic burden for society. We therefore consider long-term sickness absence to be a relevant outcome for evaluating the performance of a mental health instrument used in population surveys.

The two questionnaires, the MDI and the MHI-5, have different designs (see Table 1): the MDI questions are a list of specific symptoms of depression; the MHI-5 questions are more generally formulated. Despite the differences, both instruments have been validated as measures of depression [12,13] and both have been shown to predict sickness absence [4,5]. In the present study we compare the instruments in the same study in a so-called head-to-head comparison, and we are therefore able to identify the best performing instrument in regard to the prediction of long-term sickness absence. We also divide the population into high risk and low risk groups by several methods for each instrument, and calculate the prediction of long-term sickness absence using different categorisations of the scales.

**Table 1:** The Mental Health Inventory (MHI-5) and the Major Depression Inventory (MDI)

**MHI-5**		**Response categories**
	How much of the time during the last 4 weeks, have you….	
(1)	Been a very nervous person?	(a) All of the time
(2)	Felt so down in the dumps that nothing could cheer you up?	(b) Most of the time
(3)	Felt calm and peaceful?	(c) A good bit of the time
(4)	Felt downhearted and blue?	(d) Some of the time
(5)	Been a happy person?	(e) A little of the time
		( f) At no time
**MDI**		**Response categories**
	How much of the time in the last 2 weeks…	
(1)	Have you felt low in spirit or sad?	
(2)	Have you lost interest in your daily activities?	
(3)	Have you felt lacking in energy and strength?	
(4)	Have you felt less self-confident?	(a) All of the time
(5)	Have you had a bad conscience or feelings of guilt?	(b) Most of the time
(6)	Have you felt that life wasn’t worth living?	(c) Slightly more than half of the time
(7)	Have you had difficulty in concentrating, e.g., when reading the newspaper or watching television?	(d) Slightly less than half of the time
(8a)*	Have you felt very restless?	(e) A little of the time
(8b)*	Have you felt subdued?	( f) At no time
(9)	have you had trouble sleeping at night?	
(10a)*	Have you suffered from reduced appetite?	
(10b)*	Have you suffered from increased appetite?	

‘*’In the MDI is only the question with the highest response value of the questions 8a/8b and of the questions 10a/10b used in the total score.

## Methods

### Study sample and design

Data was collected as part of the DAnish National working Environment Survey (DANES) from late 2008 until early 2009. The DANES was approved by the Danish Data Protection Agency, journal number: 2008-54-0553. According to Danish law, questionnaire and register based studies do not need approval by ethical and scientific committees.

Questionnaire data was collected from a random sample of the Danish employed population, age 18–59 years. Participants could answer the questionnaire either by internet or by post. Non-responders received two reminders and were finally contacted and invited to participate in a telephone interview. The study contacted 9913 persons, 6531 responded (66%), of which 4919 were employees. We excluded participants with missing values on MHI-5 and MDI (n = 439), with missing response date (n = 150), with a sickness absence spell lasting 4 or more weeks in the preceding 3 months before or overlapping baseline (n = 177), yielding a study sample of 4153 participants. In the multivariable adjusted analyses the sample was, because of missing values to covariates, further reduced to 3713 participants. The 3713 participants are 75% of the employees that returned the questionnaire. If we assume the return rate of the employees is equal to the return rate of the full sample, i.e., 66%, the sample for the final analysis is 50% of the employed people the questionnaire originally was mailed to.

Our long-term sickness absence data came from a national register, the DREAM register [14]. The DREAM register has information on all Danish social transfer payments on a weekly basis. Employers are entitled to compensation from the municipalities if an employee has an absence spell of 22 days or longer.

### The major depression inventory

The Major Depression Inventory (MDI) was developed in the late 1990s [12]. It was designed to measure depression symptoms in accordance with the symptom guidelines defined by the WHO classification for unipolar depression (ICD-10) and the American Psychiatric Association classification for major depression (DSM-IV) [12,15]. The instrument consists of 12 questions and it includes 2 algorithms that classify participants with risk of unipolar depression (mild, moderate or severe) according to the ICD-10 definition or with risk of major depression according to the DSM-IV definition. The instruments can also be used to measure depressive symptoms scored on a scale ranging from 0 to 50, a higher score indicates a higher level of depressive symptoms. Both Bech et al. [12] and Forsell [16] have validated the MDI as a measure of depression using SCAN clinical interviews.

The questions of the MDI are shown in Table 1.

We calculated the MDI’s predictive value for long-term sickness absence when we used the MDI as: (1) a standardized scale score, (2) the scale categorized into five ordinal levels [5], (3) dichotomized into likely depression or not by the cut-off ≥20 in accordance with previous studies [5,16], likely depression or not based on the DSM-IV algorithm, and likely depression or not based on the ICD-10 algorithm (we pooled the ICD-10 categories mild, moderate and severe) [12].

### The mental health inventory

The mental health inventory (MHI-5) is a five-question subscale of the general health measure SF-36 [17]. The MHI-5 includes questions referring to both positive and negative aspects of mental health, and questions referring to both depression and anxiety [18,19]. Berwick et al. [13], Cuijpers et al. [18] and Rumph et al. [20] have validated the MHI-5 as a measure for depression using clinical interviews as the gold standard. The MHI-5 has also been tested as a measure of anxiety, somatoform disorders, and substance use disorders, but has failed to perform equally well in these tests [18,20]. The questions of the MHI-5 are shown in Table 1.

We scored the MHI-5 from 0 (poor mental health) to 100 (good mental health) [21]. Several cut-offs for dividing the responders into high risk and low risk group have been suggested for the MHI-5. The cut-off points are derived from ROC-curves or through another minimum misclassification criterion. The most used cut-off point is probably 52 [4,22], other cut-off points include 54 and 74 [18] and the cut-offs 60, 68 and 76 [23]. We calculated the predictive value for long-term sickness absence using the MHI-5 as: (1) a standardized scale score; (2) categorized into 5 ordinal levels; and (3) dichotomized by four different cut-offs, i.e. the cut-offs 52, 60, 68 and 76.

### Long-term sickness absence

Our outcome was time until onset of first episode of long-term sickness absence. In our analysis, long-term sickness absence was defined as minimum 4 weeks of consecutive sickness absence, because 4 weeks are the minimum period that is registered within the weekly based DREAM register. We followed responders in the register from 12 weeks before response date and until 60 weeks after response date. Participants, with long-term sickness absence in the 12 weeks before or overlapping the particular participant’s response date, were excluded from the study. Only sickness absence that comes after the response of the questionnaire is included in our analyses.

### Covariates

The following covariates were included in the analyses: Age, gender, family status, smoking, alcohol, body mass index, leisure time physical activity, social class, somatic chronic illness, self-rated health and method of data collection. With the exception of age and gender, which were retrieved from registers, the information on the covariates came from the questionnaire.

We divided family status into four categories: (1) cohabitating, with children living at home, (2) cohabiting, without children living at home, (3) not cohabitating, with children living at home, (4) not cohabiting, without children living at home. Smoking was categorized into four levels; (1) never smoked, (2) ex-smoker, (3) light smoker, i.e., from occasional smoker to 14 cigarettes a day, and (4) heavy smoker ≥15 cigarettes a day. Alcohol intake was categorized into four levels following the guidelines of the Danish National Board of Health: (1) ≤ 7 units weekly, (2) 8–14 units, (3) 15–21 units, (4) > 21 units weekly. Body mass index was categorized into 4 levels according to the WHO categorization: (1) < 18.5 (underweight), (2) 18.5–25 (normal weight), (3) 25–30 (overweight), (4) > 30 (obese). Leisure time physical activity was scored on a scale from 0 to 24 points. Social class was categorized in accordance with the European Socio-economic Classification (ESeC). Somatic chronic illness was constructed as a binary variable (somatic chronic illness or not). Self-rated health was scored on a scale from 1–5 points. The self-rated health question primarily measures physical health [24,25] and is therefore used together with somatic chronic illness to control for the responders physical health. Method of data collection was included, since responses are influenced by the data collection method [26]. Family status and social status were used as categorical variables in the analyses. All other covariates were assumed to be ordinal or linear.

### Statistical analyses

We compared the MDI and MHI-5 in a head-to-head design [27]. We calculated the Pearson correlation between the MDI and the MHI-5 and we compared the distribution of MDI and the MHI-5 in two plots. We calculated the predictive value for long-term sickness absence by Cox’s proportional hazard in both a univariate regression analysis and a multivariate regression analysis. Participants were censored at the date of emigration, death, retirement, maternity leave or end of follow-up (60 weeks after baseline), whichever came first. We examined the proportional hazard assumption by visual inspection of log-log-survival plots. The predictive value of MDI and MHI-5 for long-term sickness absence was calculated with (1) measures dichotomized by different cut-offs, (2) 5-level categorical indexes, and (3) standardized scores. We standardized the two scores of the MDI and MHI-5 using mean = 0 and standard deviation = 1. The standardized MHI-5 score was reversed so high score equalled poor mental health for both scores. In order to evaluate which mental health instrument that had the best prediction of long-term sickness absence, we included both standardized scores in the same regression analysis. If one measure in such an analysis have a substantial better significance level than the other measure, it can be concluded that this measure better capture the variation in the data. To further confirm the result from this analysis, we performed a cross-validation by randomly splitting our sample into two independent samples and then carry out the analysis in each sample.

## Results

### The MDI

The MDI standardized score, the five-level categorized score, and the three dichotomized scores all predicted long-term sickness absence (see Table 2). The standardized score had the highest significance level. The 5-level categorization of MDI showed a steep increase in HR from the next highest to the highest level. This suggests that the best cut-off point for a dichotomization into a risk group and non-risk group is near this score, i.e., near 20 points (risk group = 7%). The risk group included 90 to 301 persons depending on which dichotomization we used, i.e., the risk group was from 2 to 7% of the employed adult population (see Table 2).

**Table 2:** The MDI’s and the MHI-5’s predictive value for long-term sickness absence

			**Univariate**		Adjusted ‘*’	
		**N**	**HR [CI 95]**	**p-value**	**HR [CI 95]**	**p-value**
MDI		4153	1.44 [1.31; 1.59]	3.31E-14	1.27 [1.12; 1.43]	0.0001
MHI-5		4153	1.50 [1.35; 1.66]	9E-15	1.37 [1.21; 1.55]	5.57E-07
MDI (with MHI-5 as covariate)					1.07 [0.92; 1.26]	0.37
MHI-5 (with MDI as covariate)					1.31 [1.11; 1.54]	0.001
MDI dichotomized						
	MDI cutpoint 20 depression (yes/no)	301	2.79 [2.00; 3.88]	1.5E-09	1.98 [1.36; 2.89]	0.0004
	MDI ICD-10 depression (yes/no)	113	3.58 [2.29; 5.60]	2.28E-08	2.08 [1.26; 3.43]	0.004
	MDI DSM-IV depression (yes/no)	90	3.40 [2.05; 5.64]	2.23E-06	1.92 [1.08; 3.41]	0.03
MDI categorical						
	MDI = 0–4 (reference level)	1591	1		1	
	MDI = 5-9	1456	1.55 [1.11; 2.17]		1.42 [0.99; 2.03]	
	MDI = 10-14	515	2.33 [1.57; 3.45]		1.60 [1.03; 2.50]	
	MDI = 15-19	290	2.05 [1.25; 3.38]		1.38 [0.80; 2.38]	
	MDI > =20	301	4.08 [2.74; 6.07]		2.68 [1.68; 4.27]	
	(p-value for all 5 levels)			8.52E-11		0.001
MHI-5 dichotomized						
	MHI cutpoint 52 mental ill (yes/no)	304	2.58 [1.84; 3.62]	4.32E-08	1.92 [1.32; 2.81]	0.0007
	MHI cutpoint 60 mental ill (yes/no)	595	2.85 [2.17; 3.73]	2.82E-14	2.20 [1.62; 2.99]	4.83E-07
	MHI cutpoint 68 mental ill (yes/no)	978	2.44 [1.90; 3.14]	4.82E-12	1.99 [1.49; 2.66]	2.83E-06
	MHI cutpoint 76 mental ill (yes/no)	1560	2.05 [1.59; 2.64]	2.14E-08	1.57 [1.18; 2.09]	0.002
MHI-5 categorical						
	MHI = 91–100 (reference level)	1104	1		1	
	MHI = 81-90	1093	1.02 [0.67; 1.54]		1.01 [0.65; 1.57]	
	MHI = 71-80	978	1.39 [0.94; 2.07]		1.21 [0.78; 1.86]	
	MHI =61-70	383	1.77 [1.10; 2.87]		1.57 [0.94; 2.61]	
	MHI < 60	595	3.41 [2.35; 4.94]		2.56 [1.67; 3.92]	
	(p-value for all 5 levels)			5.75E-13		9.09E-06

‘*’The adjusted analyses include the covariates: sex, age, family status, smoking, alcohol, body mass index, leisure time physical activity, social class, somatic chronic illness, self-rated health and data collection method.

### The MHI-5

The MHI-5 standardized score, the five-level categorized score, and the four dichotomized scores all predicted long-term sickness absence (see Table 2). The standardized score had the highest significance level. The 5-level categorization of MHI-5 showed an increase in HR for each level. The increase between the next highest and the highest level of the categorization was particular steep. This suggests that the best cut-off point for a dichotomization is near 60 points (risk group = 14%). The risk group included from 304 to 1560 persons depending on which dichotomization we used, i.e., from 7 to 38% of the employed adult population.

### MDI and MHI-5 compared

The HRs of the standardized scores estimate the increased risk for one standard deviation increase of the standardized score, i.e., the increased risk of sickness absence for a one standard deviation of worse mental health. In the adjusted analyses, the HR of MDI was 1.27 and the HR of MHI-5 was 1.37, both were highly significant (see Table 2). With both instruments included in the same regression analysis, the MHI-5 remained significant and the MDI became non-significant (see Table 2), i.e., the MHI-5 explained a significant part of the variation in long-term sickness absence even when we controlled for the MDI. To confirm the stability of this result, we cross-validated the result by randomly splitting our sample in two independent samples and then carry out the analysis in each sample. Both results supported our initial finding (results not shown). Thus, the MHI-5 explained the variation in long-term sickness absence better than the MDI.

**Figure 1:**
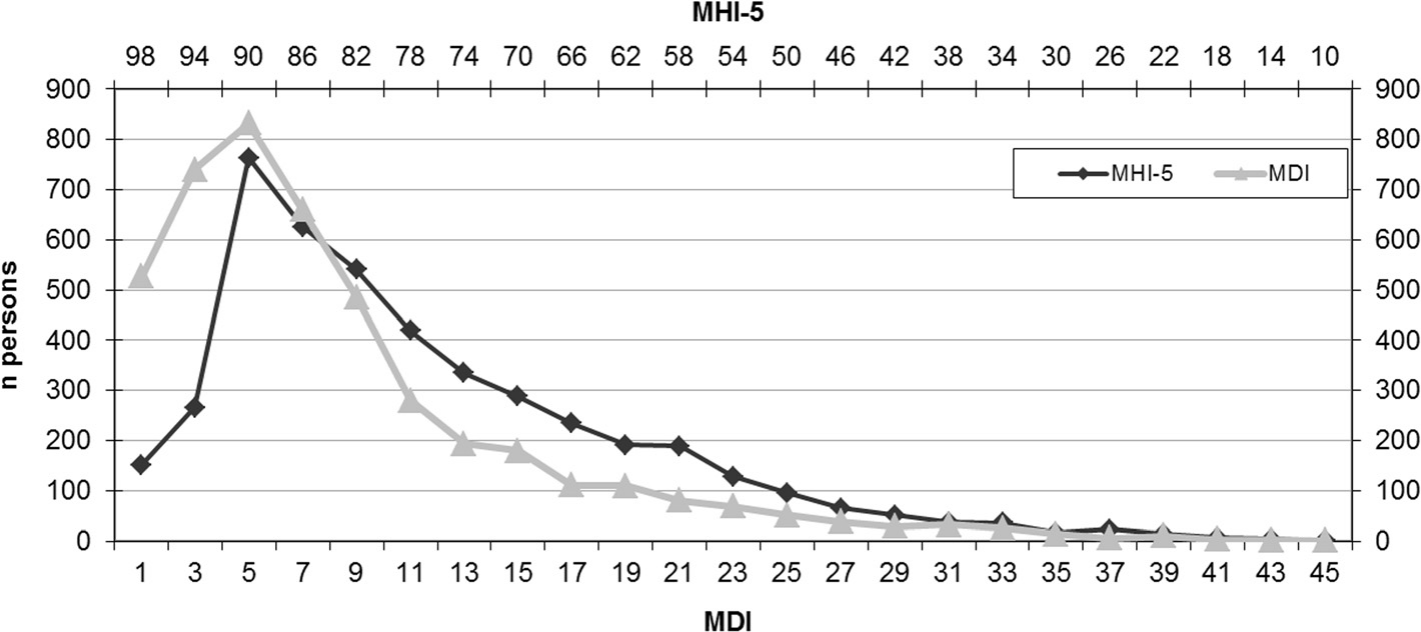
The distribution of MDI and MHI-5 in a random sample of Danish employees.

**Figure 2:**
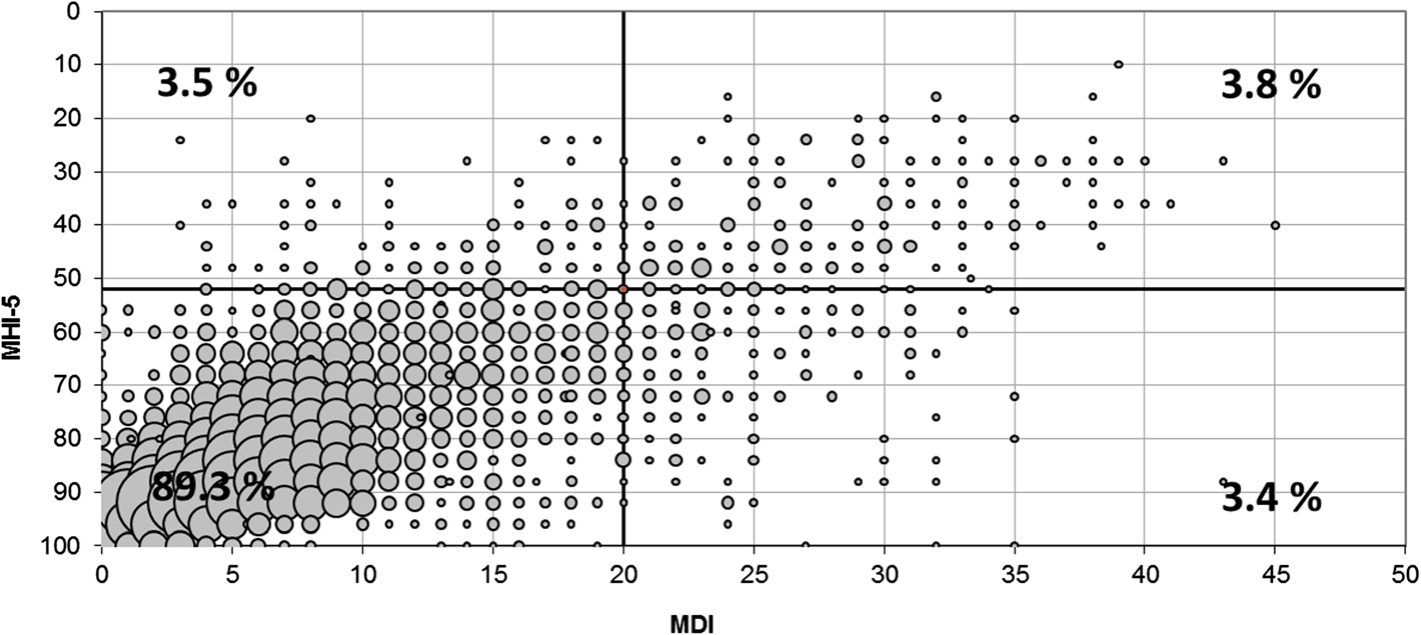
**The association between MDI and MHI-5.** Bubble size indicates no. of persons with the particular response pattern. The MDI cut-off at 20 and the MHI-5 cut-off at 52 are marked with a line, the resulting cross-hair illustrates that ‘cases’ identified by MDI and MHI-5 is only partly overlapping.

Figure 1 shows the distribution of MDI and MHI-5 scores within the study population. Both distributions are positively skewed, indicating that they distinguish better between degrees of reduced mental health than degrees of good mental health. The MDI score correlated with the MHI-5 score with a Pearson correlation coefficient = −0.69 and p < 0.0001. The correlation is illustrated in Figure 2. The Figure also illustrates that not all participants classified as high risk by the MDI were classified as high risk by the MHI-5 or vice versa. The MDI ‘cut-off 20’ and the MHI-5 ‘cut-off 52’ each classified 7.3% as high risk. However, only 3.8% of the ‘cases’ overlapped.

## Discussion

Both the standardized MDI score and the standardized MHI-5 score strongly predicted long-term sickness absence. However, the MHI-5 captured more of the variability than the MDI when both instruments were included in the same analysis. The MHI-5 therefore performs better than the MDI as a predictor of long-term sickness absence. Furthermore, the MHI-5 consists of fewer questions than the MDI and is therefore more ‘economic’. Our study can, however, not conclude that MHI-5 is a better measure of depression than the MDI; it can only conclude that the predictive value for long-term sickness absence is higher for the MHI-5 than for the MDI.

The size estimations of the high risk group from the different dichotomizations of the MDI (2 to 7%) were more restrictive than the size estimations of the high risk group from the different dichotomizations of the MHI-5 (7 to 38%). If the MDI only identified people with risk of depression and the MHI-5 identified people with reduced mental health in general, the MDI should indeed identify less people than the MHI-5. This could also explain why the MHI-5 is a better predictor of sickness absence than the MDI. However, studies show that the MHI-5 performs better as a measure of depression than as a measure of anxiety or substance disorder [13,18,20,28]. It is possible that the MHI-5 is more general than the MDI but the MHI-5 is not general enough to show strong validity as a measure of other disorders than mood disorder.

We chose to compare the predictive validity (i.e., the ability to predict a relevant outcome) of the two instruments. A limitation of our study is that only one outcome was tested, time until long-term sickness absence. Other outcomes, such as number of sick days or productivity at the workplace, may have given a different result. The population, in which we test the instruments, may also be of importance for the result. The MDI might perform better than the MHI-5 in a high risk population. The population in our study is a general working population, and only approximately 50% of those the questionnaire was aimed at ended up in the final regression analyses. The final sample may have been particular healthy due to selection bias. Furthermore, long-term sickness absence can be caused by other reasons than reduced mental health. It could, as an example, be due to a somatic illness. We have tried to control for physical health in the analyses, but it may not be sufficient.

## Conclusion

The MHI-5 had a higher predictive value for long-term sickness absence than the MDI. In a study where the predictive value for long-term sickness absence is of importance the MHI-5 must be recommended as the best measure of mental health. The size of the high risk group can for the same instrument be very different depending on the choice of cut-off for case-ness, however, the MDI categorized in general fewer persons as ‘cases’ than the MHI-5.

## Abbreviations

MDI: Major depression inventory; MHI-5: Mental health inventory; DANES: DAnish National working Environment Survey; HR: Hazard ratio.

## Competing interest

We would like to declare the following conflict of interest: The author Jakob Bue Bjorner is currently employed by QualityMetric that owns the rights to SF-36, of which the MHI-5 is a subscale. The remaining authors declare that they have no conflicts of interests.

## Authors’ contributions

SVT and JBJ were involved in the data collection. SVT, RER came up with the original idea to the manuscript. SVT, RER, JBJ and PUH all contributed to the design/focus and the discussion of results. SVT carried out the statistical analysis and prepared the manuscript. All authors read and approved the final manuscript.

## Pre-publication history

The pre-publication history for this paper can be accessed here:

http://www.biomedcentral.com/1471-2288/13/115/prepub

## References

[B1] Olesen J Gustavsson A Svensson M Wittchen HU Jonsson B The economic cost of brain disorders in Europe Eur J Neurol 2012 19 155 162 10.1111/j.1468-1331.2011.03590.x 22175760

[B2] Gamperiene M Nygard JF Sandanger I Lau B Bruusgaard D Self-reported work ability of Norwegian women in relation to physical and mental health, and to the work environment J Occup Med Toxicol 2008 3 8 10.1186/1745-6673-3-8 18430207 PMC2373783

[B3] Kaewboonchoo O Saleekul S Usathaporn S Factors related to work ability among Thai workers Southeast Asian J Trop Med Public Health 2011 42 225 230 21323186

[B4] Bültmann U Rugulies R Lund T Christensen KB Labriola M Burr H Depressive symptoms and the risk of long-term sickness absence: a prospective study among 4747 employees in Denmark Soc Psychiatry Psychiatr Epidemiol 2006 41 875 880 10.1007/s00127-006-0110-y 16951921

[B5] Hjarsbech PU Andersen RV Christensen KB Aust B Borg V Rugulies R Clinical and non-clinical depressive symptoms and risk of long-term sickness absence among female employees in the Danish eldercare sector J Affect Disord 2011 129 87 93 10.1016/j.jad.2010.07.033 20797794

[B6] Stansfeld SA Fuhrer R Head J Impact of common mental disorders on sickness absence in an occupational cohort study Occup Environ Med 2011 68 408 413 10.1136/oem.2010.056994 21075767 PMC3095482

[B7] Lerner D Adler DA Rogers WH Chang H Lapitsky L McLaughlin T Work performance of employees with depression: the impact of work stressors Am J Health Promot 2010 24 205 213 10.4278/ajhp.090313-QUAN-103 20073388 PMC4174367

[B8] Spee H Smits N de Koning H The usefulness of the Kessler Psychological Distress Scale (K10) for estimating the prevalence of depression and anxiety disorders Tijdschrift voor gezondheidswetenschappen 2012 90 145 149

[B9] Rukavina TV Brborovic O Fazlic H Dzakula A Cusa BV Prevalence and five-year cumulative incidence of psychological distress: the CroHort study Coll Antropol 2012 36 Suppl 1 109 112 22338757

[B10] Caron J Fleury MJ Perreault M Crocker A Tremblay J Tousignant M Prevalence of psychological distress and mental disorders, and use of mental health services in the epidemiological catchment area of Montreal South-West BMC Psychiatry 2012 12 183 10.1186/1471-244X-12-183 23110632 PMC3549723

[B11] Olsen LR Mortensen EL Bech P Prevalence of major depression and stress indicators in the Danish general population Acta Psychiatr Scand 2004 109 96 103 10.1046/j.0001-690X.2003.00231.x 14725589

[B12] Bech P Rasmussen NA Olsen LR Noerholm V Abildgaard W The sensitivity and specificity of the major depression inventory, using the present state examination as the index of diagnostic validity J Affect Disord 2001 66 159 164 10.1016/S0165-0327(00)00309-8 11578668

[B13] Berwick DM Murphy JM Goldman PA Ware JE Barsky AJ Weinstein MC Performance of A 5-item mental-health screening-test Med Care 1991 29 169 176 10.1097/00005650-199102000-00008 1994148

[B14] Hjollund NH Larsen FB Andersen JH Register-based follow-up of social benefits and other transfer payments: accuracy and degree of completeness in a Danish interdepartmental administrative database compared with a population-based survey Scand J Public Health 2007 35 497 502 10.1080/14034940701271882 17852980

[B15] Olsen LR Jensen DV Noerholm V Martiny K Bech P The internal and external validity of the major depression inventory in measuring severity of depressive states Psychol Med 2003 33 351 356 10.1017/S0033291702006724 12622314

[B16] Forsell Y The major depression inventory versus schedules for clinical assessment in neuropsychiatry in a population sample Soc Psychiatry Psychiatr Epidemiol 2005 40 209 213 10.1007/s00127-005-0876-3 15742226

[B17] Ware JE Jr Kosinski M Bjorner JB Turner-Bowker DM Maruish M SF-36 Health Survey. Manual and Interpretation Guide 2007 2 QualityMetric Incorporated: Lincoln, RI

[B18] Cuijpers P Smits N Donker T ten Have M de Graaf R Screening for mood and anxiety disorders with the five-item, the three-item, and the two-item mental health inventory Psychiatry Res 2009 168 250 255 10.1016/j.psychres.2008.05.012 19185354

[B19] Yamazaki S Fukuhara S Green J Usefulness of five-item and three-item mental health inventories to screen for depressive symptoms in the general population of Japan Health Qual Life Outcomes 2005 3 48 10.1186/1477-7525-3-48 16083512 PMC1201161

[B20] Rumpf HJ Meyer C Hapke U John U Screening for mental health: validity of the MHI-5 using DSM-IV Axis I psychiatric disorders as gold standard Psychiatry Res 2001 105 243 253 10.1016/S0165-1781(01)00329-8 11814543

[B21] Ware JE Jr Snow KK Kosinski M Gandek B The sf-36 Health Survey 1993 Boston: The Health Institute, New England Medical Center

[B22] Holmes WC A short, psychiatric, case-finding measure for HIV seropositive outpatients: performance characteristics of the 5-item mental health subscale of the SF-20 in a male, seropositive sample Med Care 1998 36 237 243 10.1097/00005650-199802000-00012 9475477

[B23] Kelly MJ Dunstan FD Lloyd K Fone DL Evaluating cutpoints for the MHI-5 and MCS using the GHQ-12: a comparison of five different methods BMC Psychiatry 2008 8 10 10.1186/1471-244X-8-10 18284689 PMC2265280

[B24] Bjorner JB Kristensen TS Orth-Gomér K Tibblin G Sullivan M Westerholm P Self-rated health: a useful concept in research, prevention and clinical medicine 1996 Stockholm: Swedish Council for Planning and Coordination of Research

[B25] Thorsen SV Burr H Diderichsen F Bjorner JB A one-item workability measure mediates work demands, individual resources and health in the prediction of sickness absence Int Arch Occup Environ Health 2012 [Epub ahead of print] 10.1007/s00420-012-0807-z 22922770

[B26] Feveile H Olsen O Hogh A A randomized trial of mailed questionnaires versus telephone interviews: response patterns in a survey BMC Med Res Methodol 2007 7 27 10.1186/1471-2288-7-27 17592653 PMC1925106

[B27] Kosinski M Keller SD Ware JE Hatoum HT Kong SXD The SF-36 Health Survey as a generic outcome measure in clinical trials of patients with osteoarthritis and rheumatoid arthritis-relative validity of scales in relation to clinical measures of arthritis severity Med Care 1999 37 MS23 MS39 10.1097/00005650-199905001-00003 10335741

[B28] Strand BH Dalgard OS Tambs K Rognerud M Measuring the mental health status of the Norwegian population: a comparison of the instruments SCL-25, SCL-10, SCL-5 and MHI-5 (SF-36) Nord J Psychiatry 2003 57 113 118 10.1080/08039480310000932 12745773

